# Anti-Vascular Endothelial Growth Factors as a Potential Risk for Implant Failure: A Clinical Report

**DOI:** 10.1155/2020/6141493

**Published:** 2020-02-01

**Authors:** Elham Emami, Pierre de Grandmont, Mélanie Menassa, Nicholas Audy, Robert Durand

**Affiliations:** ^1^Faculty of Dentistry, McGill University, Montreal, QC, Canada; ^2^Faculty of Dentistry, Université de Montréal, Montreal, QC, Canada; ^3^Private Clinic, Saint-Jérôme, QC, Canada

## Abstract

Knowledge of the risk factors for implant osseointegration is essential for clinical decision-making and optimizing treatment success. This clinical report presents a rare case of implant failure in a patient who received intravitreal injections of a vascular endothelial growth factor (VEGF) inhibitor for the treatment of age-related macular degeneration. Following CARE guidelines, the report presents a case rehabilitated with a mandibular 2-implant overdenture using the immediate-loading protocol and standard procedures. The implants failed within six weeks of immediate loading although primary stability (≥50 Ncm) was achieved during surgery and clinical follow-ups did not show any deviance from standard implant care or patient-related complications. Further investigation suggested that the intake of a VEGF inhibitor may be the cause of failure. This clinical report highlights the importance of systemic risk factors in implant success and their consideration during planning for implant-assisted treatment.

## 1. Introduction

Osseointegration is defined as the “process whereby clinically asymptomatic rigid fixation of alloplastic materials is achieved and maintained in bone during functional loading” [1]. A predictable outcome of any bone interaction is dependent on the maintenance of living status of bone. Therefore, angiogenesis, which is the outgrowth of new capillary blood vessels from the preexisting vessels by migration and proliferation of endothelial cells, is an essential process during both intramembranous and endochondral bone formation, bone healing, and osseointegration of implants [2, 3].

Vascular endothelial growth factor (VEGF) is a growth factor involved in many human physiologic processes such as angiogenesis [4]. VEGF is a key component of neovascularization and plays a crucial role in the restoration of vascular bone supply during the bone healing process [4, 5]. Following implant placement in the bone and the initiation of the clotting process, the platelets release several cytokines and growth factors. These factors attract the inflammatory cells and mediate the chemotactic response. Several studies have shown the effectiveness of VEGFs on bone formation and bone tissue engineering models [6, 7]. Thus, any medication that inhibits VEGFs could potentially hinder bone healing and osseointegration. However, evidence remains scarce on osseointegration pharmacology and the impact of medication on osseointegration. Thus, a knowledgeable and expert clinician may expose an individual to some consequences or harm, due to the absence of any systematically developed, evidence-based guidelines. In order to respect the duty of care, assure quality of care, and meet the demands of third-party agencies and regulatory bodies, any possible harm or side effects or unexpected therapeutic failures should be reported, investigated, and assessed rapidly.

## 2. Case Report

Following CARE guidelines for case reports [8], this clinical report presents the case of an atypical implant failure that occurred during a clinical trial conducted at Université de Montréal, Oral Health and Rehabilitation Research Unit. The study was approved by the Université de Montréal Ethics Board, and informed consent was obtained from all participants. The results of this trial concerning the immediate loading of a two-implant unsplinted mandibular overdenture and the details of clinical procedures have been published previously [9]. In brief, following standard prosthodontic and surgical procedures, all study participants received a new set of maxillary and mandibular complete prosthesis (before the surgical phase) and three threaded implants (OsseoSpeed™, Dentsply Implants, Mölndal, Sweden) using an immediate-loading (within 24 hours of surgery) protocol on two of the three implants.

**Figure 1:**
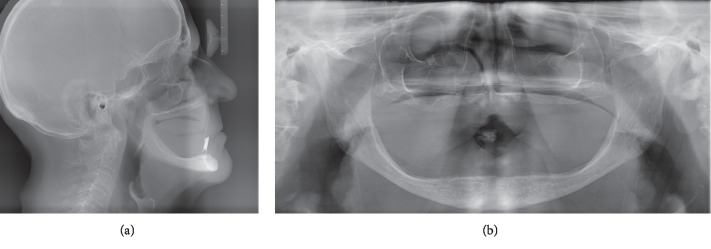
(a) Preoperative cephalometric radiograph. (b) Preoperative panoramic radiograph.

**Figure 2:**
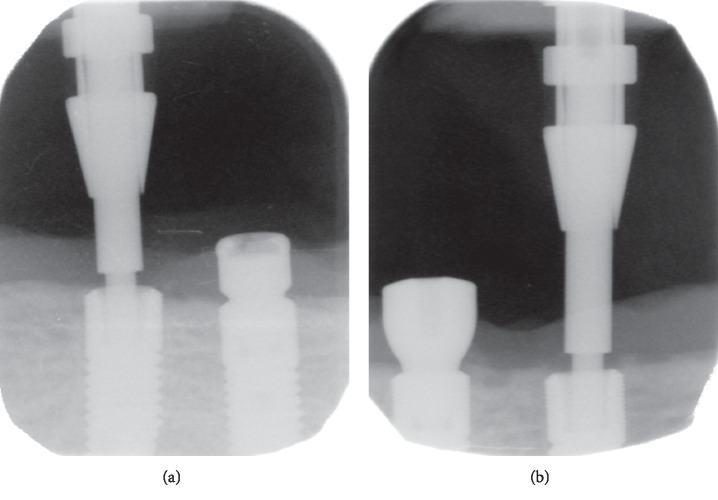
Standardized periapical radiograph taken immediately after (a) implant placement #43 and (b) implant placement #33.

**Figure 3:**
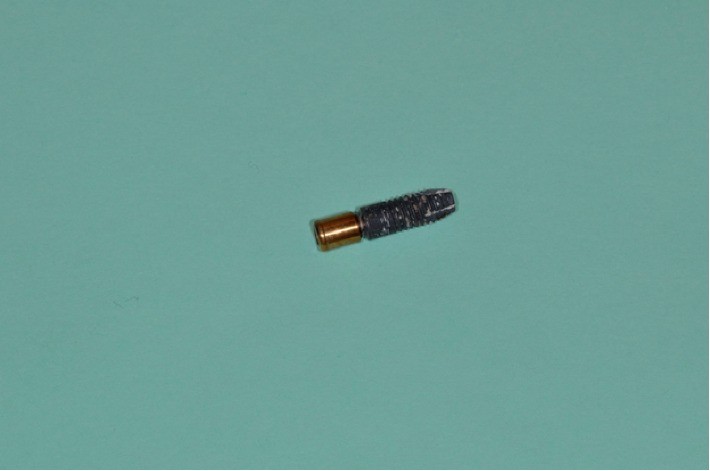
Implant loss.

The connection of right- and left-side implants and prostheses was established via unsplinted abutments (Locator® abutment, ZEST Anchors L.L.C., Escondido, CA, USA). The midline implant was unloaded for within-patient comparison on peri-implant bone crest height and implant stability. The opposing maxillary dentition for all patients was rehabilitated with a conventional removable complete denture. Of the 18 participants, one participant lost the left implant because of parafunctional habit. Another patient who experienced implant loss (female, 76 years old), and who is the subject of this case report, lost both loaded implants. Implant loss occurred within 6 weeks of immediate loading. The medical and dental history and panoramic and cephalometric preoperative radiographs (see Figures 1(a) and 1(b)) as well as clinical examinations had not revealed any contraindication for insertion of implants and immediate-loading protocol. The patient had a knife-edge mandibular edentulous ridge with an anterior bone height of 13 mm and bone width at a midheight of 7 mm. For both implants, primary stability was achieved and initial torque values were all above 50 Ncm (see Figures 2(a) and 2(b)). After implant failure (Figure 3), the patient's medical history was reviewed again, and at that time, the patient informed the research team that she had been taking an intraocular injection of Ranibizumab (Lucentis®, Genentech USA Inc., South San Francisco, CA, USA) every two months for about a year, for the treatment of age-related macular degeneration (AMD). She specified that 20 days before implant placement, she had received an injection. The mandibular overdenture of the patient was converted into a single-implant overdenture by placing a Locator® anchor system on the midline implant. To date, i.e., 7 years after implant placement, the patient has not reported any complication with her prosthesis.

## 3. Discussion

This clinical report suggests a possible impact of VEGF inhibitors on implant osseointegration. VEGF inhibitors such as Ranibizumab are used routinely for the treatment of age-related macular degeneration (AMD) [10, 11]. AMD is a disease associated with aging. It is a leading cause of adult irreversible blindness in developed countries and the third cause of worldwide blindness [12–14]. AMD has two major types: nonexudative or atrophic or “dry” AMD and neovascular or exudative or “wet” AMD. The wet form of AMD is characterized by choroidal neovascularization, the growth of abnormal blood vessels from the choroid underneath the macula [15]. Ranibizumab binds to VEGFs and inhibits VEGF-dependent angiogenesis and decreases vascular permeability [16]. As a consequence, this medication has the potential to have a negative impact on osseointegration. However, the extent to which an intravitreal injection of Ranibizumab might have systemic side effects remains unclear and should be addressed in future studies.

Despite distinctive clinical relevance, knowledge about the potential effect of inhibiting VEGF-dependent angiogenesis in dental implant osseointegration is limited. Mair et al. studied the impact of TNP-470, an inhibitor of angiogenesis, on the peri-implant bone formation and osseointegration [17]. Their results showed a negative effect and highlighted the need to investigate the effect of VEGF inhibitors on osseointegration. The findings of our recent in vivo animal study supported this clinical report hypothesis. In this study, the effects of VEGF inhibitors on bone healing and implant osseointegration were examined in the rat tibia [18]. The bone-implant contact percentage, angiogenesis, and bone defect volume were assessed by the use of histology, histomorphometry, and micro-CT analysis. The results showed that in rats that received anti-VEGF neutralizing antibody or Ranibizumab, bone-implant contact percentage was lower than in the control group, at the level of statistical significance. Furthermore, this treatment inhibited the formation of new blood vessels and diminished the blood vessel density. The size of the bone defect was significantly larger in the anti-VEGF group.

On the contrary, the potential impact of immediate loading on implant failure should not be ignored, especially since both immediately loaded implants failed while the midline control implant healed uneventfully. It was initially recommended, in the conventional oral implant-loading protocol for rehabilitation with a mandibular overdenture, that loading of the implant should occur after a minimum of 3 months of healing after implant placement in the mandible, to let the implant osseointegrate with the surrounding bone [19]. In recent years, with the development of new roughened implant surfaces, the literature has recommended immediate- or early-loading protocols in the mandible to improve the quality of life of edentate patients [20]. A recent meta-analysis on this topic showed similar implant success rates for immediate- or early-loading protocols when compared to the conventional loading protocol for mandibular implant overdentures [21]. In addition, the implant short-term success rate has been reported to not be associated with the number of implants or the design of suprastructure (splinted versus freestanding) [22]. Nevertheless, immediate exposure of the two implants to masticatory forces could be a potential cause for the bone resorption surrounding both implants. Therefore, we can conclude that the peri-implant bone submitted to an immediate loading might have been more susceptible to being affected by residual concentrations of Ranibizumab. In fact, as previously highlighted by Esposito et al. [23], it is not possible to predict implant failures. A clinical report contributes to scientific development in the preliminary stages of an investigation since it suggests hypotheses, which may be tested systematically from bedside to the bench and then, from laboratory experiments to large clinical trials, and finally to clinical guidelines. Further investigation on osseointegration and medication via collaborative research and merging data from several sources is recommended.

## 4. Conclusion

Implants may fail for different reasons that are not always easy to determine. The intake of Ranibizumab, a commonly used VEGF inhibitor, may introduce a risk for implant success due to its negative effects on bone formation and osseointegration. Data from large clinical cohorts are needed for further investigation on this topic.
